# Data verification at health centers and district health offices in Xiengkhouang and Houaphanh Provinces, Lao PDR

**DOI:** 10.1186/1472-6963-14-255

**Published:** 2014-06-14

**Authors:** Vanphanom Sychareun, Visanou Hansana, Alongkone Phengsavanh, Kongmany Chaleunvong, Ko Eunyoung, Jo Durham

**Affiliations:** 1 Faculty of Postgraduate Studies, University of Health Sciences, Vientiane, Lao PDR; 2 Faculty of Basic Sciences, University of Health Sciences, Vientiane, Lao PDR; 3 WHO Office, Vientiane, Lao PDR; 4 School of Population Health, Australia, University of Queensland, Herston, Brisbane, Australia

**Keywords:** Data verification, Indicators, Health information system, Lao PDR

## Abstract

**Background:**

Routine health information is an essential health system building block. In low and low-middle income countries however, concerns about the quality of routine administrative data have often undermined their use. The purpose of the present study was to verify the data availability, and consistency of six key maternal and child health indicators (first antenatal care, fourth antenatal care, skilled birth attendants, postnatal care, ‘Bacillus Calmette Guerin and diphtheria-pertussis-tetanus third dose).

**Methods:**

The study collected data for the identified indicators in 2011 from Xiengkhouang and Houaphanh provinces in the Lao People’s Democratic Republic (PDR). The data came from health centres (N = 109), sub-districts (N = 26) and district health offices (N = 16). Core indicators were calculated using numerators and denominators from the different data sources at the district and health centre level and standardized statistical tests performed.

**Results:**

The study revealed that data for the six indicators were either not available or not complete in the service logbooks or registers in most of the health centres. Furthermore, few health centres kept the data for up to five years, often destroying it once the report had been sent to the district health office. In addition, there was limited numerator consistency between the different data sources.

**Conclusion:**

Data on the six indicators collected and reported in the public health system across the two provinces lacked completeness, accuracy and consistency. To improve the quality of data, there is a need to train health centre staff in data collection and recording as well as ensuring there is adequate monitoring and supervision. A uniform national standardized form is also necessary with findings shared with district health offices and centres. Additionally, staff should be encouraged to own and value local data.

## Background

Improvements in maternal and child health are universally accepted health system goals. Reliable data on key maternal and child health (MCH) indicators at national and sub-national levels are essential in tracking progress of interventions designed to improve MCH. As such, a key health system building block is the Health Information System (HIS). An effective HIS generates information which enables managers to identify the health problems and make evidence-based decisions to inform investment decisions [1]. For HIS to be useful however, complete, accurate and timely reports of data from primary health care facilities up to the central level are essential. Studies in low/lower-middle income countries however have frequently observed that health data are incomplete, inaccurate and not timely [2]. Problems constraining HIS performance include: data quality [3,4]; data use [5]; poor data analysis [4]; and suboptimal HIS management practices [6]

Lao People’s Democratic Republic (Lao PDR) is a low-middle income country situated in South East Asia. The country and has a population of about 5.9 million, most of which remains rural. Maternal and under five child mortality is improving but remains unacceptably high (357/100 000 and 79/1000, respectively) [7]. The health system is predominantly public and has four administrative strata: central, provincial, district and health centre level with a strong vertical structure. There is a limited private sector, based mainly in the larger urban areas with approximately 1993 private pharmacies, 222 private cclinics and 600 traditional medicine practitioners [8]. Recently, six private hospitals have opened in the country. While decentralized, in practice the Ministry of Health continues to play a central administrative role although the provincial and district departments of health level are key actors in service delivery at health facilities and community level [8]. At the local level if health staff see that their coverage rates are below the target set by the central government, they have the authority to take steps to improve coverage rates.

In common with many other health systems in low-middle income countries, the health system is under-resourced, has poor geographical reach and low productivity [9]. Making informed investment decisions to improve the health system however, first requires reliable data that reflects the processes of care and clinical outcomes. To begin to address this, the facility-based Lao Health Management Information System (HMIS) was introduced and is compulsory by decree [10]. By the end of 2008, most health institutions in the country had been provided training in the HMIS 2004 instruments and the HMIS had been rolled out in almost all provinces [10].

**Figure 1:**
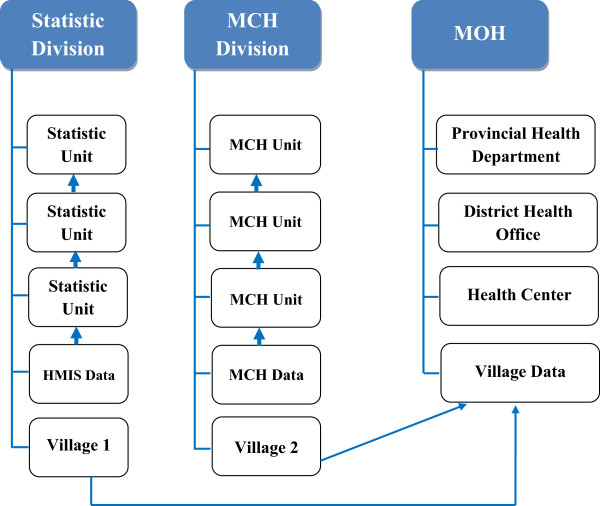
Diagram of Vertical data collection system (OR Facility-based and village-based data collection system).

The HMIS is divided into 17 provincial health departments (PHDs). These are further subdivided into district health offices and health centres. At the village level implementation is optional depending on available resources [10]. The reporting chain flows from the health centres to the district health office, from the district health office to the provincial health department and from provincial health office to central level (MOH) (See Figure 1). Most of the data is completed using a paper based system with just over half of the district level staff being able to use a computer to consolidate data from health centre and district hospital [10]. Both facility-based and outreach services are recorded on the same registers [10].

There are significant challenges in effective implementation of the HMIS including lack of human resources and the capacity for supervision and monitoring of data collected by different vertical programs [11]. In addition, in terms of MCH and Expanded Program of Immunization (EPI) while data should be integrated into one national MCH and EPI form, the HMIS data is collected by the district statistics unit, while the MCH and EPI data are collected by district MCH units with limited communication between the two units [11]. Another challenge is the absence of accurate denominators used to calculate the coverage rate especially for maternal and child health. For example, the denominator used for the estimated number of children live birth, children under 1 year, children under 5 years, pregnant women, and women age 15–49 is based on 2005 census [7]. However, this estimation does not account for cross-border migration or family planning programs. As a result, there is no formal confirmation that the estimated target population used for the calculation of indicators is accurate [7]. For these reasons we wanted to assess the completeness and accuracy of key Maternal, Newborn and Child Health (MNCH) indicators against which data is routinely collected and reported. The purpose was to first verify the availability of data for six core MNCH indicators (ANC1, ANC4, SBA delivery, PNC, BCG, and DPT3) based on health centre and district level monthly reports in Xiengkhuang and Houaphan provinces. Second, we wanted to evaluate the consistency of the six core MCH indicators at the health centre and district health levels through comparison of data from the different sources, including village-based data, EPI-MCH and HMIS. Finally, we wanted to calculate and compare the six MCH indicators by using different sources of numerators and denominators.

## Methods

### Setting

The study setting was two Northern provinces: Xiengkhouang and Houaphanh. These provinces were selected because the provincial health system had been implementing the integrated MNCH program in collaboration with Ministry of Health with technical and financial support from WHO since 2010. The population of Xienhkounag is 249,817. Phonesavanh is the capital city and there are eight district health offices/district hospitals and 55 health centres in Kham, Khoune, Morkmay, Nonghet, Pek, Phaxay, Phoukoud, and Thatom districts. In addition, there are 18 sub-districts which are directly covered by district hospitals and not by a health center. Houaphanh province has a population of about 280,000. Xamneua is the capital and there are eight districts: Xamneua, Xiengkhor, Viengthong, Viengxay, Huameuang, Xamtay, Ead, and Sopbao. It has eight district health offices/district hospitals and 54 health centres with 8 sub-districts. All 109 health centres and 26 sub-districts in the sixteen districts were included in the assessment, resulting in total of 135 health centres/sub-districts. In both provinces, rurality and poor transport infrastructure make accessibility at the district and village difficult, especially in the rainy season. The health centres in both provinces are completing the second health microplan which details the activities for 2012, having submitted the first health microplan in 2011 that was introduced by MoH/WHO as a part of the MNCH program. The monitoring table in the microplan includes a table to be completed to demonstrate the coverage rates of the core MCH indicators for the year and targets for the following year.

### Definition of the six MCH indicators and data sources from 2009 to 2011

For the purpose of the present study, six MCH indicators were assessed. Table 1 provides the definition of these. Family planning indicators were excluded. This was because while the number of IUDs, condoms and contraceptive pills distributed can be counted, calculating coverage is problematic in the absence of a registration book to follow up every couple. For the family planning indicator, it is suggested that monitoring through a survey process every five years will yield more accurate results.

**Table 1:** Definition of main six MCH indicators

**Indicator**	**Definition**	**Numerator**	**Source**	**Denominator**	**Source**
ANC1	Percentage of pregnant women attended, at least once ANC visit during their pregnancy, by skilled health personnel for reasons relating to pregnancy.	No of pregnant women attended, at least once ANC visit during their pregnancy, by Skilled health personnel.	VHV	No of pregnant women in its catchment area in the given year from 1^st^ Jan to 31^st^ Dec (If not available, it could be calculated by [Number of births x 1.05])	VHV
			MCH-EPI		MCH-EPI
			HMIS		HMIS
			Projection		Projection
ANC4	Percentage of pregnant women who received her fourth antenatal care in the given month either in the facility or during outreach	No of pregnant women who received her fourth antenatal care in the given month	VHV		VHV
			MCH-EPI		MCH-EPI
		(Note: Please do not count those who received fifth or more antenatal care)			
			HMIS		HMIS
			Projection		Projection
PNC1	Percentage of mothers who received her first postnatal care within 7 days after birth in the given month either in the facility or during outreach	No of mothers who received her first postnatal care within 7 days after birth in the given month either in the facility or during outreach	VHV	No of childbirths: Number of women who gave births in its catchment area in the given year from 1^st^ Jan to 31^st^ Dec (Note: Not total number of newborns in case of stillbirths or twins)	VHV
			MCH-EPI		MCH-EPI
			HMIS		HMIS
		(Note: Please do not double count those who received second or more postnatal care)	Projection		Projection
Delivery with SBA	Proportion of all expected births that take place in public health facilities or during outreach under the supervision of a trained professional or Skill Birth Attendant.	No of all expected births that take place in public health facilities or during outreach under SBA.	VHV	Number of childbirths: Number of women who gave births in its catchment area in the given year from 1^st^ Jan to 31^st^ Dec (Note: Not total number of newborns in case of stillbirths or twins)	VHV
			MCH-EPI		MCH-EPI
			HMIS		HMIS
			Projection		Projection
BCG	Percentage of children who received BCG vaccination in the given month either in the facility or during outreach	No of children who received BCG vaccination in the given month either in the facility or during outreach.	VHV	Number of infant (under 1 year): Number of infant (under 1 year old) in its catchment area at certain time of the given year (such as 1^st^ Jan or middle of year or 31^st^ Dec)	VHV
			MCH-EPI		MCH-EPI
			HMIS		HMIS
			Projection		Projection
DPT-HepB3	Percentage of children who have received at least three doses of DPT-HepB vaccine in the first year of life either in the facility or during outreach	No of children who have received at least three doses of DPT-HepB vaccine in the first year of life (Note: Please do not count those who received DTP1 or DTP2).	VHV	No of infant (under 1 year): Number of infant (under 1 year old) in its catchment area at certain time of the given year (such as 1^st^ Jan or middle of year or 31^st^ Dec)	VHV
			MCH-EPI		MCH-EPI
			HMIS		HMIS
			Projection		Projection

A detailed description of each source of numerator and denominator is provided in Table 2. For the numerators, there are three different data sources: VHV data, MCH-EPI data and HMIS data. For the denominators, there are three data sources: VHV data, administrative data and projections based on the 2005 population census.

**Table 2:** Data sources

**Numerator**	**Source**	**Description**
	VHV data	Village-based data collected by VHVs. Central MCH Center and WHO have implemented this in the two provinces in this study. It is planned to integrate this level of data into HMIS.
	MCH/EPI data	Facility-based data collected by health centers or hospitals. Health centers report this to the MCH district health unit with the MCH Unit reporting to the provincial MCH unit. It is slightly different from HMIS and more specific to MCH. The central MCH Center has used this data for many years, but recently announced that it would stop using this form. However, some provincial and district health office still use this.
	HMIS data	National facility-based data collection composed of 4 parts-inpatient, outpatient, MCH and financial report. Health centers report this to statistics unit of the district health offices and the district statistic unit report to the provincial statistic unit.
**Denominator**	**Source**	**Description**
	VHV data	Village-based data collected by VHV. Central MCH Center and WHO implemented in the two provinces in this study. It is planned to be integrated into HMIS.
	Administrative data	Administratively collected data by village head reported to district administrative offices. Then shared with health centers and district health offices. It does not include the number of pregnant women, but includes the number of children <1 and the number of births.
	Census projection	An estimated projection of the population up to 2015, based on the 2005 census. It includes number of children < 1 year and the number of births. The number of pregnant women is not estimated and at times, this is again calculated by multiplying the number of birth by 1.1.

### Data collection

Eight graduate researchers from the Faculty of Postgraduate Studies, University of Health Sciences made up the research team. The team was provided a preliminary, two-day training from the principal investigator (VS) in collaboration with the MoH and WHO. Data collection took place between 30^th^ April and 30^th^ May 2012, with only 2011 data included. One team member was assigned to two districts and each member then visited every health centre in their assigned district. Each district has on average seven-eight health centres, meaning each investigator spent approximately 14 days in each of their assigned districts. In collecting the data, the researchers worked closely with the district staff. This included spending an additional two days working at the district office with the officer responsible for HMIS and the MCH statistics officer in order to compile the data into a standardised excel sheet as well as making copies of health centre data for verification. Once the numerator and denominator data was entered, the researchers and district staff could automatically produce graphs to compare the data from the different sources.

### Data analysis

The coverage rate of the six core MCH indicators was calculated by making combinations of the numerators and denominators from the different sources as defined in Table 3. Simple descriptive statistics were produced using Microsoft Excel 2007.

**Table 3:** Availability of data from different sources at the health center levels for six main MCH indicators

**Indicator**	**2011**		
	**Availability**	**Logbook**	**Monthly report**
ANC1	99.8% (1618/1620)	32% (515/1618)	68%) (1013/1618)
ANC4	99.8% (1618/1620)	32% (515/1618)	1103/1618 (68%)
SBA	100% (1620/1620)	24% (384/1620)	76% (1168/1620)
PNC1	95.8% (1552/1620)	25% (384/1552)	75% (1168/1552)
BCG	97.7% (1584/1620)	80% (1272/1584)	20% (252/1524)
DPT3	97.7% (1583/1620)	82% (1295/1583)	18% (288/1523)

Note: Numerator is calculated as [(Monthly data x12 months x number of health centers & sub-districts from 2 provinces (135) = 1620)] –[missing value of monthly data during 12 months from all Health centers and Subdistricts from 2 provinces].

Denominator is calculated as [monthly data of each health center x12 months x number of Health Centers & sub-districts (135) = 1620) from 2 provinces].

Data from the monthly report is data from HC monthly reports.

Data from the logbook is the data registered from the logbook of health center.

#### Data availability

To assess the availability of monthly data in the health centres, the research team checked for the absence or presence of the numerators of the six main indicators in the logbooks. Ideally, the log books should be kept at the health centre and on monthly basis, health centre staff should fill out the report form and then send to district health office. One copy of the report should be kept in health centre so that they can refer to it whenever it is needed. In cases where there was no raw data in the logbooks, the researchers checked the monthly report from health center submitted to the district health office. Health centre reports should clearly differentiate missing data from zero values. A true zero value indicates that no reportable events occurred that month, whereas a missing value indicates that reportable events occurred but were not actually reported. Data reporting for a specific MCH indicator was considered complete if a value for the element was available for each month of the study period 2011, especially the numerators of the six main MCH indicators. At the district level, the researchers checked for the absence or presence of the numerators of the main six MCH indicators from the HMIS and EPI-MCH sources which were reported from the health centres and compiled at the district level, before being sent to the provincial health office.

#### Data consistency

We assessed two types of data consistency: numerator and denominator consistencies. To assess consistency of the numerators, we compared the consistency of the numerators of the six main indicators (ANC1, ANC4, SBA, PNC, BCG, DPT3-Hep) from the health centre logbooks with the HMIS and MCH-EPI data from district health facilities. Consistency analysis of the denominators was performed by dividing the estimated denominators from the projected 2005 census by the actual denominators collected by VHVs. This is defined as the consistency ratio. The closer the consistency ratio is to 1 (or 100%), the higher the consistency [12,13]. A consistency ratio of less than .67 was taken to denote too low a projection and a consistency ratio of >1.33 to indicate too high a projection [12,13].

#### Calculation the coverage of six MCH indicators

The researchers calculated the coverage of the six MCH indicators by using different numerators (health centre, HMIS and EPI-MCH) and different denominators (VHV, administrative data, and projections).

#### Comparison of six MCH indicators with the gold standard

To compare the coverage of the six core MCH indicators, the researchers used the more reliable numerators from the logbooks and the denominators from the VHV’s data collection. Then, we calculated the consistency ratio by dividing the coverage from the logbooks and VHVs with coverage from district health facility records (HMIS and EPI-MCH) for six MCH indicators. A similar cut off point was considered for the interpretation of the consistency ratio of the six MCH indicators. That is, a consistency ratio of less than .67 was taken to denote too low a projection and a consistency ratio of >1.33 to indicate too high a projection [12,13].

### Ethical approval

The study protocol was approved by the University of Health Sciences Ethical Committee for Health Research, Lao PDR. In addition, the local authorities and the Provincial Department of Health in Xiengkhouang and Houaphanh provinces approved the study. All the data was de-identified, routine in nature and collected with the intent of eventually becoming publicly available, thus individual consent was not required.

## Results

### Data availability

Availability of indicators refers to the extent to which health centre monthly reports included all reportable events from each health centre during 2011 for the six selected MCH indicators (ANC1, ANC4, SBA, PNC1, BCG, and DPT3-Hep). Missing values at the health center level were observed. This was especially observed for PNC1 which had a higher number of missing values than the other MCH indicators. Only a small percentage of data from the logbooks was available but most of the data was filed and available in the monthly reports. As Table 3 indicates about 24% of SBA and 25% of PNC data were derived from the logbook compared to immunization indicators (Table 3).

### Data consistency

#### Consistency of data numerators

To check the consistency of data between the data obtained from the health centres and district health offices, we compared the numerators from the three different sources to find out the matching numerator data of the different sources. Differences in the numerators were observed by source for the VHVs, HMIS and MCH-EPI. The numerators from HMIS and MCH-EPI however were almost the same as seen in Table 4. Numerators from the health centers on the other hand, demonstrated a large difference compared to data from the HMIS and MCH-EPI sources. The data from the health centre was higher than the data from HIMS and MCH-EPI for ANC1, ANC4, SBA, indicators, but the numerators of PNC, BCG and DPT3 indicators from the health centres were lower than that from HIMS and MCH-EPI.

**Table 4:** Percentage agreement of numerator data for MCH indicators 2009, 2010 and 2011, district level

	**No of agreement for two sources**	**No of agreement for three sources**
**Indicator**	**2011**	**2011**
ANC1	5 (31.25%)	2 (12.5%)
ANC4	5 (31.25%)	1 (6.25%)
SBA	7 (43.7%)	1 (6.25%)
PNC1	6 (37.5%)	1(6.25%)
BCG	5 (31.25%)	2 (12.5%)
DPT3	6 (37.5%)	1 (6.25%)

Note: Unit analysis is district health facility.

Zero values and missing data were excluded from the calculation of the percentage of agreement of numerators.

Numerator: Number of district health facilities having the same numerators with the data compiled from Health centers and from HMIS or EPI-MCH.

Denominator: Total number of district health facilities surveyed.

We also calculated the proportion of district health offices in which the data were identical and the percentage of agreement between two and three different sources. Of the 18 district figures which matched exactly, 7.5% of the aggregated district reports for measles immunization and 31.25% of aggregated district reports for ANC1 matched the available information from the HMIS and MCH-EPI over the 12-month period in 2011 (Table 4). Most of the agreements were from four district facilities in Xiengkhouang province: Pek, Phaxay, Mok and Thathom districts. In other words, discrepancies in the data were observed in the majority of the health facilities.

### Consistency of denominators

#### Consistency of estimated number of child births

The consistency ratio of the estimated number of childbirths is the estimation of the number of childbirths divided by the actual number of childbirths collected by the VHV. In total there were 11 districts with a consistency ratio of >1.33 (68.7%) demonstrating that the projection was too high. One district revealed a consistency ratio of < .67 (6.3%) indicating too low a projection. Almost all of the estimated number of childbirths was too high when compared with the actual number of childbirths. Information related to the denominator from the different sources is presented in Table 5.

**Table 5:** Denominators from different sources by districts of the Xiengkhouang and Houaphanh provinces

	**No pregnant women**	**Consistency ratio**	**No childbirth**	**Consistency ratio**	**No infant < 1 year**	**Consistency ratio**
**Xiengkhouang province**						
Health Center	1782	1.17	1507	1.32	1398	1.37
EPI-MCH	1920	1.09	1514	1.31	1497	1.28
HMIS	1920	1.09	1514	1.31	1497	1.28
Projection	2086		1987		1914	
**Huaphanh province**						
Health Center	819	2.05	780	2.05	741	2.06
EPI-MCH	819	2.05	780	2.05	741	2.06
HMIS	819	2.05	780	2.05	741	2.06
Projection	1680		1600		1525	

Note:

Consistency ratio of Denominators = Projected Denominator/Existing Denominator at each level of health facility.

Denominator: Number of actual pregnant women from Health Center, EPI-MCH, HMIS and projection.

Consistency ratio of number of pregnant women at the health center in Xiengkhouang =2086/1782 = 1.17.

#### Consistency of estimated number of pregnant women

The number of pregnant women was calculated based on the number of childbirths multiplied by 1.05. We used two sources of estimations that is, from the VHVs and from the population census. Consistency of the estimated number of pregnant women is the estimation of the number of pregnant women divided by the actual number of pregnant women collected by the VHVs. The analysis revealed six districts with a consistency ratio >1.33 suggesting too high a projection. In addition, one district with a consistency ratio < .67 indicated too low a projection in 2011. The actual number of pregnant women recorded by the VHVs and the projected number of pregnant women varied, as the projected number of pregnant women was higher than the actual number of pregnant women recorded by the VHVs. Two districts (Xamtai and Sobbao) used the same sources of denominators as the health centres, that is, EPI-MCH and HMIS.

#### Consistency of estimated number of child under one year old

The number of children under one year old varied by district and by the different sources of data collection. For example, the number of children under one year was higher than the projection compared to the actual number of children under one year old. Consistency of the estimated number of children under one year old is defined as the estimation of the number of children under one year old divided by the actual number of children under one year old by the VHV. There were four districts with a consistency ratio >1.33 for which the projection was too high. In addition, one district revealed a consistency ratio of < .67 indicating too low a projection.

### Consistency for coverage rate

The calculation of the six main indicators is dependent on the accuracy of the numerators of the events and the denominators. There are three different sources of numerators and denominators that is, administrative health centre data (from registers or monthly reports), EPI-MCH, and HMIS. The denominators from the administrative can be considered unreliable, as the source of denominator was unknown. The MCH indicators were therefore calculated using the different sources of numerators and denominators to examine which was the most valid. Differences between the six main MCH indicators and the EPI-MCH, HMIS and the health center data were observed. If the data from health centres and VHVs were accurately detecting all health care service delivery and provided a good estimation of the population denominators, the values of indicators derived from different sources would be similar with the HC and VHVs. As seen in Table 6, the coverage from the denominator VHV and the numerators from HMIS and MCH-EPI were somewhat similar to the gold standard. The lowest coverage for all MCH indicators was found for the numerator from HMIS and MCH-EPI and the denominator projection.

**Table 6:** Six MCH Indicators: Xiengkhouang and Houaphanh provinces by different sources of numerators and denominators, 2011

**Indicator**	**HC/VHV**	**HC/Adm**	**HC/Proj**	**EPI/Admin**	**EPI/VHV**	**EPI/Proj**	**HMIS/Admi**	**HMIS/VHV**	**HMIS/Proj**
**Xiengkhouang Province**									
ANC1	48.6	51.0	40.8	49.7	47.4	39.8	47.3	45.1	37.8
ANC4	35.4	37.2	29.7	35.2	33.5	28.1	34.7	33.0	27.7
SBA	21.4	22.9	14.1	27.7	25.8	17.0	24.3	22.6	14.9
PNC1	25.5	27.4	16.8	33.8	31.5	20.8	32.2	30.1	19.8
BCG	55.3	61.5	45.4	62.9	56.5	46.5	60.8	54.6	44.9
DPT3	47.2	52.5	38.8	53.9	48.5	39.9	58.3	52.4	43.1
**Houaphanh Province**									
ANC1	47.2	56.8	36.8	40.5	33.6	26.2	35.5	29.4	23.0
ANC4	25.6	30.8	19.9	23.3	19.3	15.1	31.9	26.4	20.6
	31.1	36.4	21.1	23.7	20.3	14.4	17.1	14.6	10.4
PNC1	15.0	17.5	10.6	17.9	15.3	10.9	13.3	11.4	8.1
BCG	75.9	89.4	61.2	51.7	43.9	35.4	40.0	33.9	27.4
DPT3	60.8	71.6	49.0	49.9	42.4	34.2	34.3	29.2	23.5

### Comparison of six MCH indicators with the logbooks/VHV

There is no gold standard against which to compare the indicators, so we used the numerator data from logbooks and denominators collected by VHVs as the gold standard. There were only four districts (Pek, Phaxay, Thathom and Mok districts) in which numerators were collected from the logbooks of the health centres, thus, we described only the four districts for the year 2011. If the logbook from health centres are accurately detecting all health care service delivery events and there are sound estimates of relevant population denominators from data collection of population sources by VHVs, the values for indicators derived from the HMIS and MCH-EPI should be similar to those derived from VHVs and the health centre administrative data . The logbook/VHV consistency ratio is calculated as the coverage for an indicator based on the HMIS, MCH-EPI divided by the coverage based on logbook and VHVs. The consistency ratio gives an idea of how close the intervention coverage estimated from different sources of numerators and denominators from HMIS, MCH_EPI and administrative health centre monthly reports is to the coverage obtained from logbook and VHVs data: the closer this ratio is to one (or 100%), the higher the consistency. Table 7 shows a comparison of coverage rates for antenatal care (ANC1 and ANC4) revisits, SBA, PNC, BCG and Diptheria3 from logbooks/VHVs and from health centre monthly report, HMIS, and MCH-EPI. For all of the MCH indicators, the consistency ratio was less than one for the numerators based on three different sources and from the projection of denominators. A consistency ratio of closer to one was observed among the denominators from VHVs. The consistency ratio was similar for numerators from HMIS and MCH-EPI. For ANC1 and ANC4, the consistency ratio of the numerators from HMIS and EPI-MCH and the denominator from VHV was slightly higher than one indicating higher consistency. For SBA and PNC, the consistency ratio was much higher than one, especially for the HMIS and MCH-EPI numerator data, indicating higher consistency. For BCG and DPT3 immunization coverage, the rate based on facility reports appears to be a substantial overestimate compared with the rate from the logbook. In Mok district, the consistency ratio of one was observed for numerators from HC, HMIS and MCH-EPI and denominators from HC, HMIS and MCH-EPI. For DPT3 however, the consistency ratio was less than one for the numerators from the three different sources and from the projection of the denominators.

**Table 7:** Comparison of coverage rates from logbooks/VHvs and from HMIS and MCH-EPI and consistency ratio

	**HC/VHV**	**HC/Admi**	**HC/Proj**	**EPI/Admin (CR)**	**EPI/VHV**	**EPI/Proj**	**(HMIS/Admin**	**HMIS/VHV**	**HMIS/Proj**
**Pek District**									
ANC1	61.1	56.7 (0.92)	52.1 (0.85)	57.0 (0.93)	61.4 (1.004)	52.4 (0.86)1	57.0 (0.93)	61.4 (1.004)	52.4 (0.85)
ANC4	53.9	50.0 (0.93)	46.0 (0.85)	49.9 (0.92)	53.8 (0.99)	45.9 (0.85)	49.9 (0.92)	53.8 (0.99)	45.9 (0.85)
SBA	1.8	1.8 (1.0)	1.4 (0.77)	2.5 (1.4)	2.5 (1.4)	1.9 (1.05)	2.5 (1.4)	2.5 (1.4)	1.9 (1.05)
PNC1	30.3	30.2 (0.99)	23.0 (0.76)	31.8 (1.05)	31.9 (1.05)	24.2 (0.79)	31.8 (1.05)	31.9 (1.05)	24.2 (0.79)
BCG	62.0	57.9 (0.93)	45.3 (0.73)	59.5 (0.96)	63.7 (1.03)	46.6 (0.75)	59.5 (0.96)	63.7 (1.03)	46.6 (0.75)
DPT3	44.1	41.1 (0.93)	32.2 (0.73)	41.6 (0.94)	44.6 (1.01)	32.5 (0.73)	41.6 (0.94)	44.6 (1.01)	32.5 (0.73)
**Phaxay District**									
ANC1	56.3	41.1 (0.73)	62.3 (11.1)	33.7 (0.60)	46.2 (0.82)	51.1 (0.90)	33.7 (0.60)	46.2 (0.82)	51.1 (0.90)
ANC4	25.5	18.6 (0.73)	28.3 (1.11)	20.4 (0.80)	28.0 (1.09)	31.0 (1.21)	20.4 (0.80)	28.0 (1.09)	31.0 (1.21)
SBA	28.4	25.6 (0.90)	28.0 0.99)	28.1 (0.99)	31.3 (1.10)	30.9 (1.09)	28.1 (0.99)	31.3 (1.10)	30.9 (1.09)
PNC1	32.5	29.3 (0.90)	32.1 (0.99)	20.7 (0.64)	23.0 (0.70)	22.8 (0.70)	20.7 (0.64)	23.0 (0.70)	22.8 (0.70)
BCG	77.9	64.8 (0.83)	91.9 (1.18)	58.4 (0.75)	70.3 (0.90)	82.9 (1.06)	58.4 (0.75)	70.3 (0.90)	82.9 (1.06)
DPT3	56.2	46.7 (0.83)	66.2 (1.18)	44.6 (0.79)	53.6 (0.95)	63.2 (1.12)	44.6 (0.79)	53.6 (0.95)	63.2 (1.12)
**Thathom District**									
ANC1	60.7	60.7 (1.00)	57.6 (0.95)	60.7 (1.0)	60.7 (1.00)	57.6 (0.95)	60.7 (1.00)	60.7 (1.00)	57.6 (0.95)
ANC4	36.5	36.5 (1.00)	34.7 (0.95)	36.5 (1.00)	36.5 (1.00)	33.5	36.5 (1.00)	36.5 (1.00)	33.5 (0.92)
SBA	43.6	43.6 (1.00)	31.3 (0.72)	54.3 (1.25)	54.3 (1.25)	39.0 (0.89)	54.3 (1.25)	54.3 (1.25)	39.0 (0.89)
PNC1	46.4	46.4 (1.00)	33.3 (0.72)	40.0 (0.86)	40.0 (0.86)	28.7 (0.62)	40.0 (0.86)	40.0 (0.86)	28.7 (0.62)
BCG	63.8	71.7 (1.12)	63.8 (1.00)	63.8 (0.89)	63.8 (0.89)	69.5 (1.09)	63.8 (0.89)	63.8 (0.89)	69.5 (1.09)
DPT3	71.7	71.7 (1.00)	63.8 (0.89)	78.1 (1.09)	78.1 (1.09)	69.5 (0.97)	78.1 (1.09)	78.1 (1.09)	69.5 (0.97)
**Mok District**									
ANC1	36.1	36.1 (1.00)	28.8 (0.80)	36.1 (1.00)	36.1 (1.00)	28.8 (0.80)	36.1 (1.00)	36.1 (1.00)	28.8 (0.80)
ANC4	43.9	43.9 (1.00)	35.1 (0.80)	43.9 (1.00)	43.9(1.00)	35.1 (0.80)	43.9 (1.00)	43.9 (1.00)	35.1 (0.80)
SBA	19.3	19.3 (1.00)	10.1 (0.52)	19.3 (1.00)	19.3 (1.00)	10.1 (0.52)	19.3 (1.00)	19.3 (1.00)	10.1 (0.52)
PNC1	21.1	21.1 (1.00)	11.0 (0.52)	21.1 (1.00)	21.1 (1.00)	11.0 (0.47)	21.1 (1.00)	21.1 (1.00)	11.0 (0.47)
BCG	57.7	57.7 (1.00)	40.8 (0.71)	57.7 (1.00)	57.7 (1.00)	40.8 (0.71)	57.7 (1.00)	57.7 (1.00)	40.8 (0.71)
DPT3	42.3	42.3 (1.00)	29.9 (0.70)	42.3 (1.00)	42.3 (1.00)	29.9 (0.70)	42.3 (1.00)	42.3 (1.00)	29.9 (0.70)

Note: CR-Consistency Ratio.

Numerator = coverage of the indicators from the different sources such as HC/Adm, HC/Proj, EPI/Adm, EPI/Proj, EPI/VHV, HMIS/Adm, HMIS/Proj, HMIS/Adm, HMIS/VHV.

Denominator = coverage of the indicators from HC/VHV which is considered more reliable.

Consistency ratio = coverage of the indicators from the different sources/ coverage of the indicators from source HC/VHV.

CR of ANC1 in Pek district = coverage of ANC1 from HC/Adm/ coverage of the indicators from source HC/VHV.

CR of ANC1 in Pek district: 56.7/61.1 = 0.92.

## Discussion

National health systems rely on service delivery performance data in order to allocate resources and monitor effectiveness and coverage of interventions [14]. The main purpose of the present study was to verify the availability of data for six core MNCH indicators (ANC1, ANC4, SBA delivery, BCG, DPT3 and measles) based on health centre and district level monthly reports in two provinces in the Lao PDR. The study has highlighted several deficiencies in the routine MCH service delivery data and HMIS reporting processes in these two provinces. One problem that negatively impacts on the accuracy and reliability of HMIS is that of missing data, particularly at the health centre level. This can significantly skew results to either over-report or under-report practices or outcomes of a system. Reasons for the missing or inaccurate data related mainly to health staff shortages, high staff turnover and inadequate skills. Previous studies carried out in resource-constrained environments have documented similar findings [15]. Data security was also of concern, with some health centers not performing backups regularly. Further, in general the health centers did not properly record what data was available or maintain records. Most of data for example, were destroyed after sending the report to the district health office.

In addition to missing data and poor data security, the submission dates were different for the MCH unit and HMIS unit, which resulted in different numbers being reported for the same indicators. At the health centre level an additional problem was that copies of the reports were not kept meaning the data was not readily available when needed. Further, neither the district nor the health centres, made a yearly summary which meant there was no coherent annual report. The study also found inconsistencies in the numerators between health centres and the district and between the HMIS and MCH-EPI district health office. There is some evidence to suggest that the numerators from the logbooks or registers of health centres or VHV were more reliable than the numerators from the health centre’s monthly report or the HMIS and EPI-MCH. This is because the VHV worked closely with the village leader to collect the relevant population data from their community. The denominator is too high as it is based on the 2005 population census which did not take into account migration or family planning. Given the birth rate in Lao PDR has been decreasing in the past few years it seems likely that the denominators were over rather than underestimated. It was not clear however, how denominators for EPI-MCH and HMIS were derived.

A large difference was observed between what the health centres actually submitted to the district statistic offices in the form of the monthly summary sheets, and what subsequently appeared in the HMIS and EPI-MCH. This might be due to incorrect recording of numbers, not all registers being included in the monthly report, or register data being summated before the end of the month resulting in some data not being recorded. Another explanation might be due to the lack of a standardized reporting format in the HMIS as different international funding agencies had their own report format and use different data sources. This leads to duplication and unnecessary complexity. This has been found elsewhere with difficulty of accurate data collection further complicated by different systems [15,16]. Given the importance of consistent data [2,11], agreement needs to be reached among the different stakeholders on a standardized form and the numerators and denominators to be collected. Further, given basic denominators such as the number of pregnant women, children <1 and number of births and deaths should be available from the village head and/or VHV this data should be used and the capacity of the overall system strengthened to collect this data annually rather than using estimates based on modelling and estimations. Currently however, not all the village heads and/or VHVs collect this data and further support is required. It may also be that staff at clinics do not assign significant value to the quality of data collection as they do not see that data is used to inform health services. Sharing and using the results of any MoH analysis with district health offices and health centres could contribute to increasing understanding of the value of accurate data [2]. Other studies have documented improved data quality when health staff understand the importance of accurate data and have a sense of ownership of the information generated [17,18]. A trained supervisory team at the central and provincial level could also support district health office in supervising health centre staff in data verification, calculation of coverage data and target setting [2,3]. It is also important to note that the HMIS excludes data from the private sector. There is no available data on the extent to which the private sector provides ambulatory care in the Lao PDR and while currently health care provision in Xiengkhouang and Houaphanh provinces is almost entirely through the public sector, a private sector is emerging and incorporating private sector data will become increasingly important.

As with all studies, our study has some limitations which should be taken into account. First, our study was undertaken in only two provinces and both of these were in the North. The findings therefore cannot be extrapolated beyond these two sites. Second the selected indicators have not been randomly selected as we selected based on the priorities of MCH packages. Third missing data affected our analysis. Last, we were unable to examine relationships between data completeness and its potential determinants. Nevertheless, the present paper constitutes an important contribution applicable to understanding data discrepancies in low-resourced settings.

## Conclusion and recommendation

Our results suggested that data from the health centres lack availability, completeness and consistency, which makes it difficult to monitor and supervise the service delivered at health facilities. In order to facilitate the improvement of data management, district and health centre level managers need expanded skills to enable them to evaluate the quality of their data and use it improve services. Health centre staff also need to be supported and supervised in the execution of data management tasks. Building the capacity of the HMIS is therefore a long-term project. In the meantime, and given the uncertainty about the denominator and difficulty in collecting information about the numerator, periodic household surveys which can incorporate private sector data, is likely to provide local level planners with more accurate information.

Another important issue is addressing the problem of duplication of effort. Duplication occurs when different numerators and denominators are used and there is separation between vertical and horizontal programs. In this study this situation was further complicated by the differing demands of the international agencies putting further stresses on an already under-resourced system. In accordance with a number of declarations including the Paris Declaration on Aid Effectiveness, the Accra Agenda for Action and the more recent Busan Partnership for Effective Development Cooperation, donors must coordinate and align their programs with the government’s needs and avoid duplication of effort. Finally, all units should be encouraged to own and value local data and use the HMIS to provide standardised data for indicator.

## Competing interests

The authors declare that they have no competing interests.

## Authors’ contributions

VS contributed to design the research project, data collection, and writing the preliminary report. VH and AP assisted in the study design, data collection and synthesising data. KE and KC assisted with the data collection and data analysis. KE and JD assisted with analysing and synthesizing the data. All authors read and approved the final manuscript.

## Pre-publication history

The pre-publication history for this paper can be accessed here:

http://www.biomedcentral.com/1472-6963/14/255/prepub
